# Machine learning algorithm to predict mortality in patients undergoing continuous renal replacement therapy

**DOI:** 10.1186/s13054-020-2752-7

**Published:** 2020-02-06

**Authors:** Min Woo Kang, Jayoun Kim, Dong Ki Kim, Kook-Hwan Oh, Kwon Wook Joo, Yon Su Kim, Seung Seok Han

**Affiliations:** 1 Department of Internal Medicine, Seoul National University College of Medicine, 103 Daehak-ro, Jongno-gu, Seoul, 03080 Korea; 2 Medical Research Collaborating Center, Seoul National University Hospital, Seoul, Korea

**Keywords:** Acute kidney injury, Continuous renal replacement therapy, Intensive care unit, Machine learning, Mortality

## Abstract

**Background:**

Previous scoring models such as the Acute Physiologic Assessment and Chronic Health Evaluation II (APACHE II) and the Sequential Organ Failure Assessment (SOFA) scoring systems do not adequately predict mortality of patients undergoing continuous renal replacement therapy (CRRT) for severe acute kidney injury. Accordingly, the present study applies machine learning algorithms to improve prediction accuracy for this patient subset.

**Methods:**

We randomly divided a total of 1571 adult patients who started CRRT for acute kidney injury into training (70%, *n* = 1094) and test (30%, *n* = 477) sets. The primary output consisted of the probability of mortality during admission to the intensive care unit (ICU) or hospital. We compared the area under the receiver operating characteristic curves (AUCs) of several machine learning algorithms with that of the APACHE II, SOFA, and the new abbreviated mortality scoring system for acute kidney injury with CRRT (MOSAIC model) results.

**Results:**

For the ICU mortality, the random forest model showed the highest AUC (0.784 [0.744–0.825]), and the artificial neural network and extreme gradient boost models demonstrated the next best results (0.776 [0.735–0.818]). The AUC of the random forest model was higher than 0.611 (0.583–0.640), 0.677 (0.651–0.703), and 0.722 (0.677–0.767), as achieved by APACHE II, SOFA, and MOSAIC, respectively. The machine learning models also predicted in-hospital mortality better than APACHE II, SOFA, and MOSAIC.

**Conclusion:**

Machine learning algorithms increase the accuracy of mortality prediction for patients undergoing CRRT for acute kidney injury compared with previous scoring models.

## Introduction

Acute kidney injury (AKI) is an important issue because of its related morbidities and mortality rates [1, 2]. The prevalence of AKI has been increasing by up to 50% in patients admitted to the intensive care unit (ICU) [3–5]. Continuous renal replacement therapy (CRRT) is a widely used renal replacement modality, particularly when patients have severe AKI and are unstable, because it can easily control biochemical imbalances caused by AKI [6, 7]. Despite the benefits of this modality, the mortality rate remains high, ranging from 30 to 70% [8–10]. Considering the critical condition of patients who undergo CRRT, the precise prediction of their prognosis is a topic of interest.

Several mortality prediction models for critically ill patients with AKI have been presented [11, 12]. However, these prediction models did not focus on patients requiring CRRT for AKI. Conventional scoring systems such as the Acute Physiologic Assessment and Chronic Health Evaluation II (APACHE II) and the Sequential Organ Failure Assessment (SOFA) have shown suitable performance for predicting the mortality of ICU patients [13, 14], but the predictive power appeared insufficient for CRRT patients [11]. Thus, it is necessary to introduce a new scoring model or strategy that is tailored to patients receiving CRRT.

Machine learning has been used in various clinical fields ranging in application from diagnosis to prediction [15–17]. Machine learning also appears to be useful in predicting outcomes of critically ill patients or patients with AKI [18–21]. However, machine learning algorithms have not been applied to patients undergoing CRRT for AKI. Conventional scoring models such as APACHE II and SOFA show limitations, for example, a low prediction accuracy for the CRRT subset and difficulty of adding new variables to the models. Our new abbreviated mortality scoring system for AKI with CRRT (MOSAIC model) has not been validated in other cohorts despite a high prediction accuracy of mortality for the CRRT subset [22]. Because of the success of machine learning in other clinical applications, the study explored whether machine learning algorithms are also applicable for predicting the mortality of patients initiating CRRT for AKI. The study compared the performance of several machine learning models with that of the conventional APACHE II and SOFA scores, and with the MOSAIC model.

## Methods

### Data source and study population

The study protocol complies with the Declaration of Helsinki, as revised in 2013, and was approved by the institutional review board of the Seoul National University Hospital (no. H-1903-130-1020). A total of 1610 adult patients (≥ 18 years old) who started CRRT for AKI were retrospectively reviewed at Seoul National University Hospital from June 2010 to December 2016. Patients who had underlying end-stage renal disease (*n* = 27) and those with no information on co-morbidities or laboratory data (*n* = 12) were excluded. Thus, 1571 patients were analyzed in the present study. The subjects were randomly divided into a training set (70%, *n* = 1094) to develop the models and a test set (30%, *n* = 477) to test the performance of each model.

### Study variables

Baseline characteristics such as age, sex, application of mechanical ventilation, and co-morbidities including diabetes mellitus, hypertension, myocardial infarction, chronic heart failure, stroke, peripheral vascular disease, dementia, chronic obstructive pulmonary disease, connective tissue disease, peptic ulcer disease, cancer, ischemic heart disease, chronic kidney disease, and atrial fibrillation were collected. Vital signs, such as mean arterial pressure, heart rate, respiratory rate, and body temperature, were measured at the initiation of CRRT for each patient. The laboratory data such as white blood cell count, hemoglobin, blood urea nitrogen, creatinine, albumin, pH, sodium, and potassium were measured at the time of starting CRRT. APACHE II, SOFA, and MOSAIC scores were calculated based on the calculation methods presented in the original studies [13, 14, 22]. The primary output was the ICU mortality, and the discontinuation of CRRT was censored. Information on in-hospital mortality was also collected.

### Statistical analysis

Statistical analyses were performed using R software (version 3.6.2; The Comprehensive R Archive Network: http://cran.r-project.org). Categorical and continuous variables are expressed as proportions and the means ± standard deviation, respectively. The chi-square test was used to compare categorical variables (Fisher’s exact test if not applicable). The Student’s *t* test was used to compare continuous variables. Several machine learning algorithms were used, such as κ-nearest neighbor (KNN), support vector machine (SVM), multivariate adaptive regression splines (MARS), random forest (RF), extreme gradient boost (XGB), and artificial neural network (ANN). The KNN modeling was performed using a hyperparameter selection process (κ) involving leave-one-out cross-validation to determine the best accuracy for the training set. The Euclidean distance was used to train the KNN model. The rectangular, triangular, Epanechnikov, biweight, Gaussian, rank, and optimal kernels were used in training. We developed the SVM models using various kernels including linear, polynomial, sigmoid, and radial basis functions. For each kernel, we conducted 10-fold cross-validation and selected the best hyperparameter (cost, gamma, degree, and coefficients). We selected the kernel corresponding to the highest area under the receiver operating characteristic curve (AUC) for the final SVM model. We conducted 10-fold cross-validation to develop the MARS model on the training set. The maximum degree of interaction and the MiniSpan were set to three, indicating the allowance of three evenly spaced knots for each predictor. To select the hyperparameter for the RF model, we used 10-fold cross-validation on the training set. The hyperparameter included ntree (number of trees), mtry (number of variables used in each tree), and nodesize (minimum size of nodes, which determines depth). We used 10-fold cross-validation to develop the XGB model and determined the best hyperparameter consisting of eta (step size shrinkage used in the update process to prevent overfitting), gamma (minimum loss reduction required to make a further partition), and the maximum depth of a tree. We used 10-fold cross-validation to construct the ANN model and determined the optimal hyperparameter consisting of the size (the number of hidden nodes) and decay (parameter for weight decay). When developing the KNN, SVM, and ANN models, we standardized all of the prediction variables except for the categorical variables for analysis. Herein, categorical variables were processed using one-hot encoding. Once we developed the models using the training set, we calculated the F1 score, accuracy, and AUCs on the test set to measure the performance of each model. To calculate the accuracy and F1 score of the APACHE II, SOFA, and MOSAIC results, we used the best threshold point of the receiver operating characteristic curve to determine the probability of mortality. The AUCs of the models were compared using the DeLong test. The net benefit of the machine learning model was assessed by using decision curve analysis [23, 24]. In the decision curve analysis, APACHE II, SOFA, and MOSAIC scores were converted to a logistic regression using probability theory. Calibration, which is the agreement between predicted probabilities and observed frequencies of ICU mortality, was assessed with calibration belts. All *P* values were two-sided, and values less than 0.05 were considered significant.

## Results

### Baseline characteristics

We randomly assigned 1094 and 477 patients into training and test sets, respectively. The variables remained constant between the two sets (Additional file 1: Table S1). A total of 26.7% of the patients had anuria (i.e., < 100 ml/d). The ICU and in-hospital mortality rates were similar between the training and test sets. When the patients in the training set were categorized according to the ICU mortality, most of the baseline variables differed between the groups with and without death. The APACHE II, SOFA, and MOSAIC scores were higher for the deceased patients than for surviving. Table 1 displays the other variables.

**Table 1:** Baseline characteristics of the training set

Variables	Total (*n* = 1094)	No ICU mortality (*n* = 464)	ICU mortality (*n* = 630)	*P*
Age (years)	62.6 ± 15.4	62.7 ± 15.1	62.5 ± 15.6	0.874
Male (%)	60.7	57.5	63.0	0.067
Mean arterial pressure (mmHg)	81.0 ± 17.3	82.8 ± 16.4	79.7 ± 17.8	0.003
Heart rate (/min)	105.2 ± 25.4	99.6 ± 24.2	109.3 ± 25.5	< 0.001
Respiratory rate (/min)	24.0 ± 8.4	21.9 ± 7.1	25.5 ± 8.9	< 0.001
Body temperature (°C)	36.3 ± 1.6	36.4 ± 1.4	36.3 ± 1.7	0.159
White blood cells (× 10^3^/μL)	14.1 ± 20.1	14.1 ± 11.5	14.1 ± 24.6	0.969
Hemoglobin (g/dL)	9.8 ± 2.2	10.1 ± 2.1	9.6 ± 2.2	< 0.001
Blood urea nitrogen (mg/dL)	50.3 ± 29.9	49.0 ± 29.7	51.3 ± 30.0	0.219
Creatinine (mg/dL)	2.7 ± 1.7	3.0 ± 2.0	2.5 ± 1.4	< 0.001
Albumin (g/dL)	2.8 ± 0.6	2.9 ± 0.6	2.6 ± 0.6	< 0.001
pH	7.3 ± 0.1	7.3 ± 0.1	7.3 ± 0.1	< 0.001
Sodium (mEq/L)	138.6 ± 8.1	138.3 ± 7.1	138.8 ± 8.8	0.318
Potassium (mEq/L)	4.3 ± 0.9	4.2 ± 0.8	4.4 ± 1.0	< 0.001
Target clearance (ml/min)	42.7 ± 14.1	41.8 ± 13.5	43.3 ± 14.5	0.072
Diabetes mellitus (%)	30.3	36.6	25.6	< 0.001
Hypertension (%)	27.8	29.7	26.3	0.216
Myocardial infarction (%)	8.5	10.1	7.3	0.097
Chronic heart failure (%)	15.2	17.7	13.3	0.048
Stroke (%)	12.9	15.5	11.0	0.026
Peripheral vascular disease (%)	7.8	9.1	6.8	0.174
Dementia (%)	5.2	6.9	4.0	0.031
Chronic obstructive pulmonary disease (%)	3.9	2.6	4.9	0.050
Connective tissue disease (%)	1.4	1.5	1.3	0.737
Peptic ulcer disease (%)	2.5	2.2	2.7	0.567
Cancer (%)	36.8	34.1	38.9	0.101
Ischemic heart disease (%)	12.1	14.7	10.2	0.024
Chronic kidney disease (%)	29.7	41.4	11.4	< 0.001
Ventilator apply (%)	82.9	75.2	88.6	< 0.001
Atrial fibrillation (%)	12.3	16.4	9.4	0.001
APACHE II score	35.9 ± 10.3	33.7 ± 10.4	37.6 ± 9.9	< 0.001
SOFA score	12.0 ± 3.6	10.7 ± 3.4	13.0 ± 3.4	< 0.001
MOSAIC score	20.8 ± 10.6	16.4 ± 9.2	24.1 ± 10.4	< 0.001

Data were measured at the time of initiating CRRT

*Abbreviations*: *ICU* intensive care unit, *APACHE* Acute Physiology and Chronic Health Evaluation, *SOFA* Sequential Organ Failure Assessment, *MOSAIC* Mortality Scoring system for AKI with CRRT

### Development of mortality prediction model

**Fig. 1:**
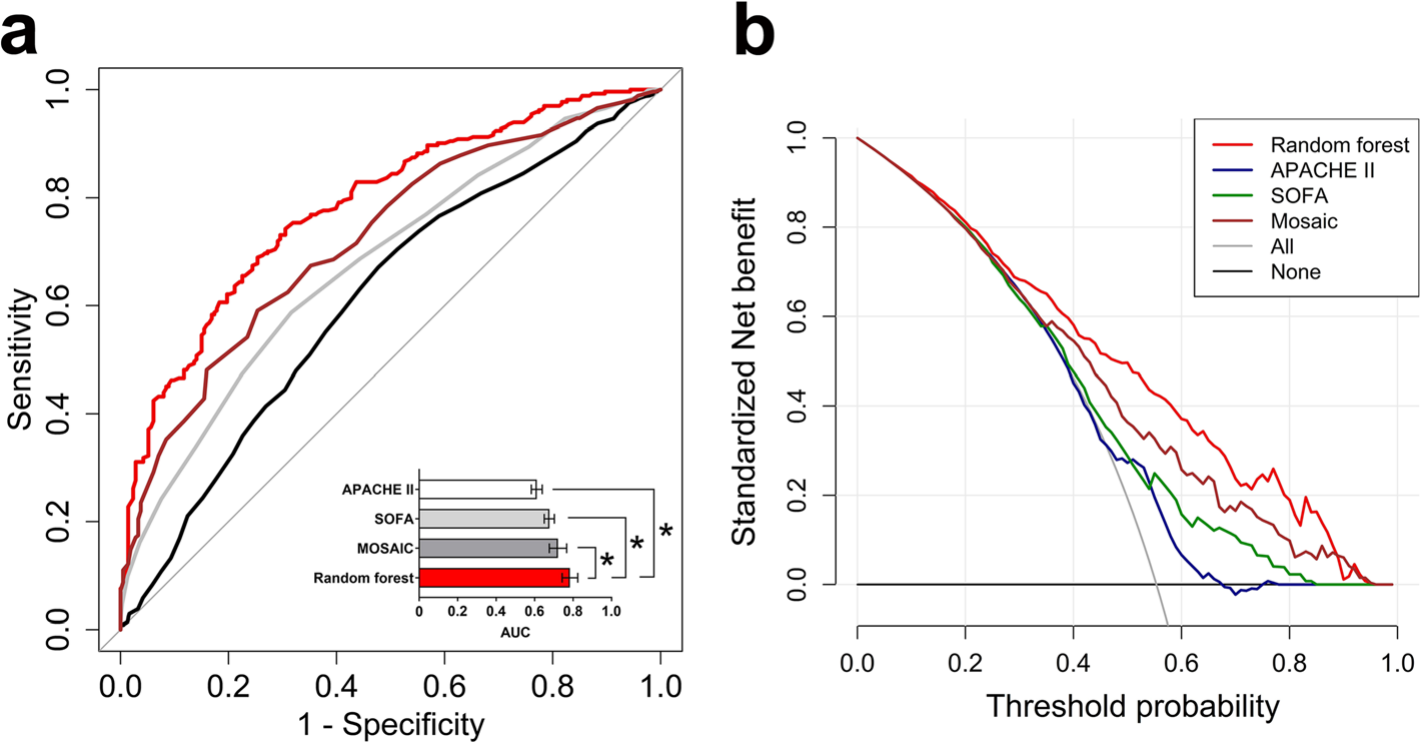
Comparisons of intensive care unit mortality prediction models such as random forest, APACHE II, SOFA, and MOSAIC in the test set. **a** Receiver operating characteristic curves of random forest, APACHE II, SOFA, and MOSAIC. The bar graph indicates the median value of the AUC in the model. The error bar indicates the range. **b** Decision curve analysis of random forest, APACHE II, SOFA, and MOSAIC. *P <  0.05. APACHE, Acute Physiology and Chronic Health Evaluation; SOFA, Sequential Organ Failure Assessment; MOSAIC, Mortality Scoring system for AKI with CRRT

A total of 894 (56.9%) patients died in the ICU. The F1, accuracy, and AUC values resulting from the test set are shown in Table 2. The AUC values of APACHE II, SOFA, and MOSAIC for the prediction of ICU mortality were 0.611 (0.583–0.640), 0.671 (0.651–0.703), and 0.722 (0.677–0.767), respectively. The AUC value of the RF model was 0.784 (0.744–0.825), which was the highest among the machine learning models. The XGB and ANN models achieved the next highest AUC value of 0.776 (0.735–0.818). The APACHE II, SOFA, and MOSAIC scores achieved lower accuracies and F1 scores than the machine learning models. The XGB models achieved the highest accuracy and F1 score. Among the machine learning models, the performance did not significantly differ, except for the difference between the RF and MARS models (Additional file 1: Table S2). The RF model demonstrated superior performance to the APACHE II, SOFA, and MOSAIC methods (*P*s <  0.05) (Fig. 1a). The better performance of the RF model than the conventional scoring systems remained consistent, even if the ICU mortality was considered without censoring the discontinuation of CRRT (Additional file 2: Figure S1). The net benefit of the RF model ranged from 7 to 95%, which was better than the ranges corresponding to the APACHE II, SOFA, and MOSAIC scores (Fig. 1b, without 95% confidence intervals [CIs]; Additional file 2: Figure S2, with 95% CIs). The machine learning models achieved better performance than the conventional scoring systems (Table 2). All of the machine learning models achieved higher F1 scores and accuracy than conventional scoring systems. The receiver operating characteristic curves of all of the evaluated models are shown in Additional file 2: Figure S3.

**Table 2:** Mortality prediction models for patients undergoing continuous renal replacement therapy in the test set

Models	AUC (95% CI)	*P* value*	*P* value^†^	*P* value^‡^	Accuracy	F1 score
APACHE II	0.611 (0.583–0.640)				0.607	0.660
SOFA	0.677 (0.651–0.703)				0.629	0.643
MOSAIC	0.722 (0.677–0.767)				0.660	0.658
κ-Nearest neighbor	0.762 (0.719–0.805)	< 0.001	< 0.001	0.213	0.673	0.745
Support vector machine	0.771 (0.729–0.813)	< 0.001	< 0.001	0.119	0.692	0.752
Multivariate adaptive regression splines	0.753 (0.710–0.796)	< 0.001	0.003	0.332	0.673	0.736
Random forest	0.784 (0.744–0.825)	< 0.001	< 0.001	0.045	0.690	0.762
Extreme gradient boost	0.776 (0.735–0.818)	< 0.001	< 0.001	0.085	0.715	0.763
Artificial neural network	0.776 (0.735–0.818)	< 0.001	< 0.001	0.082	0.694	0.749

*Abbreviations*: *AUC* area under the curve, *CI* confidence interval, *APACHE* Acute Physiology and Chronic Health Evaluation, *SOFA* Sequential Organ Failure Assessment, *MOSAIC* Mortality Scoring system for AKI with CRRT

*Compared with the APACHE II model

^†^Compared with the SOFA model

^‡^Compared with the MOSAIC model

**Fig. 2:**
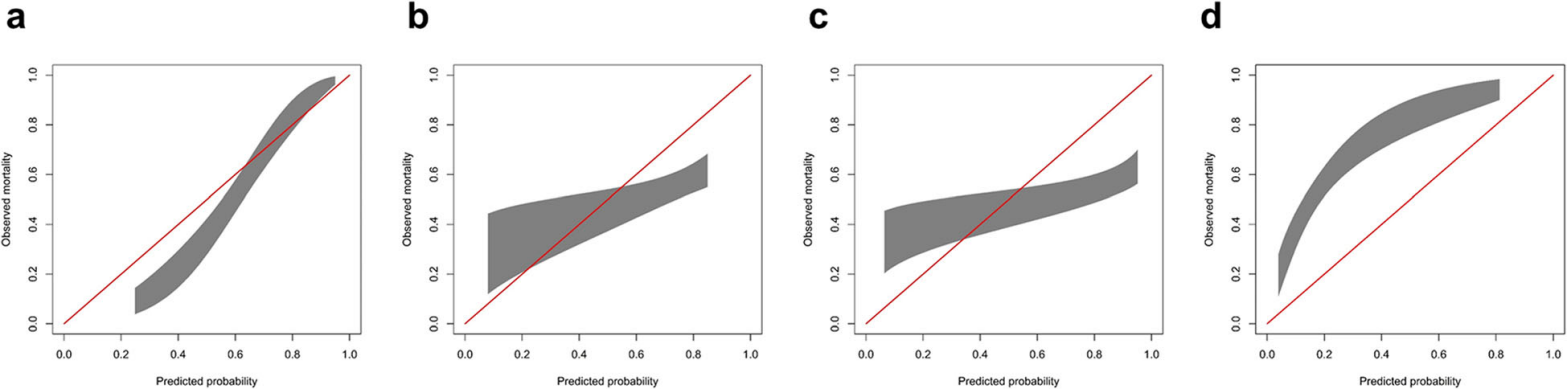
Calibration belts of **a** random forest, **b** APACHE II, **c** SOFA, and **d** MOSAIC for ICU mortality prediction in the test set

The calibration belts of the RF model and the conventional scoring systems for ICU mortality prediction are shown in Fig. 2. The RF model showed better calibration among patients at a high risk of ICU mortality than did the APACHE II, SOFA, and MOSAIC scores.

### Rank of predictors in the prediction model

**Fig. 3:**
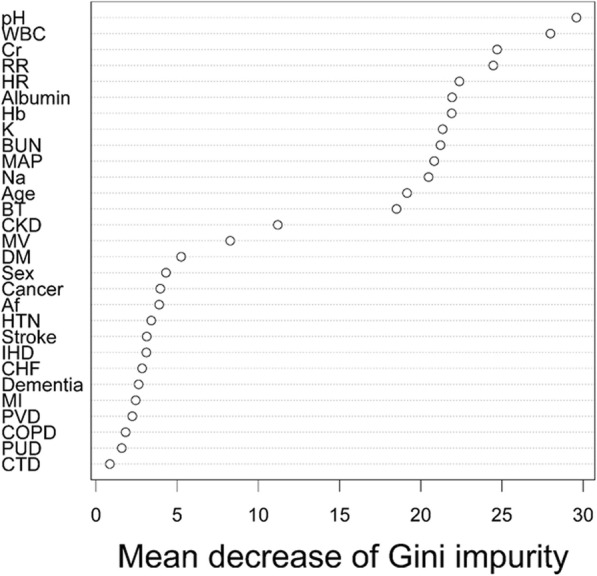
Rank of importance of variables in developing the random forest model for intensive care unit mortality prediction

**Fig. 4:**
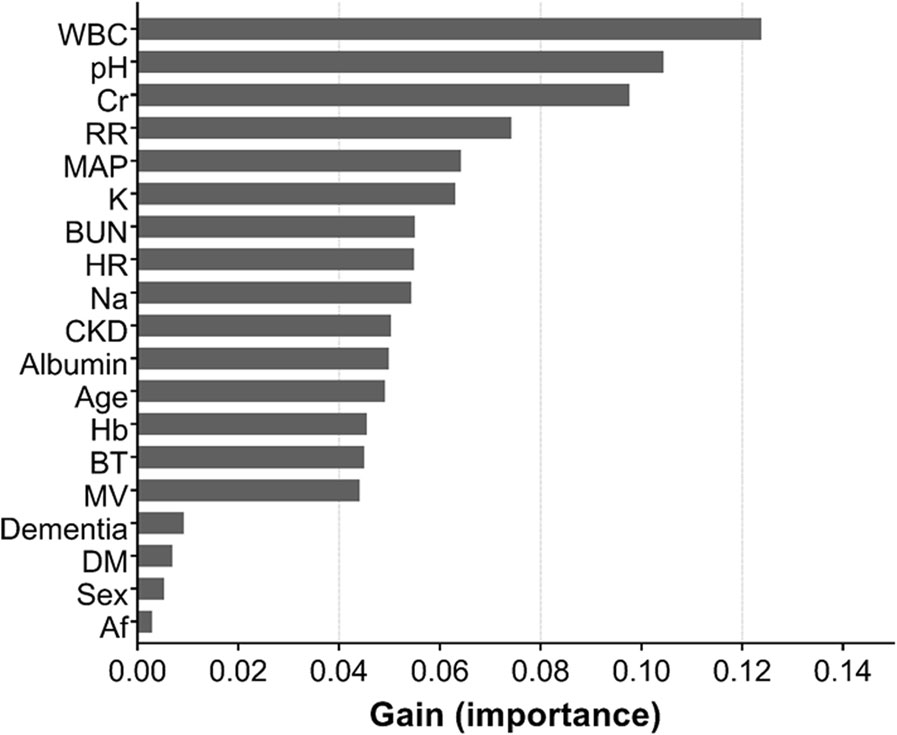
Rank of importance of variables in developing the extreme gradient boost model for intensive care unit mortality prediction

The RF model used Gini impurity to determine the variables used for the split at each node, and the mean decrease in Gini of each variable in every tree was calculated. Accordingly, the pH was the most important variable in predicting ICU mortality using the RF model, followed by white blood cells, creatinine, respiratory rate, and heart rate (Fig. 3). For the XGB model, which had the highest F1 score, the importance of variables was determined according to the sum of the decrease in error. The white blood cell count was the most important variable in predicting ICU mortality, followed by pH, creatinine, and respiratory rate (Fig. 4).

### In-hospital mortality prediction model

**Fig. 5:**
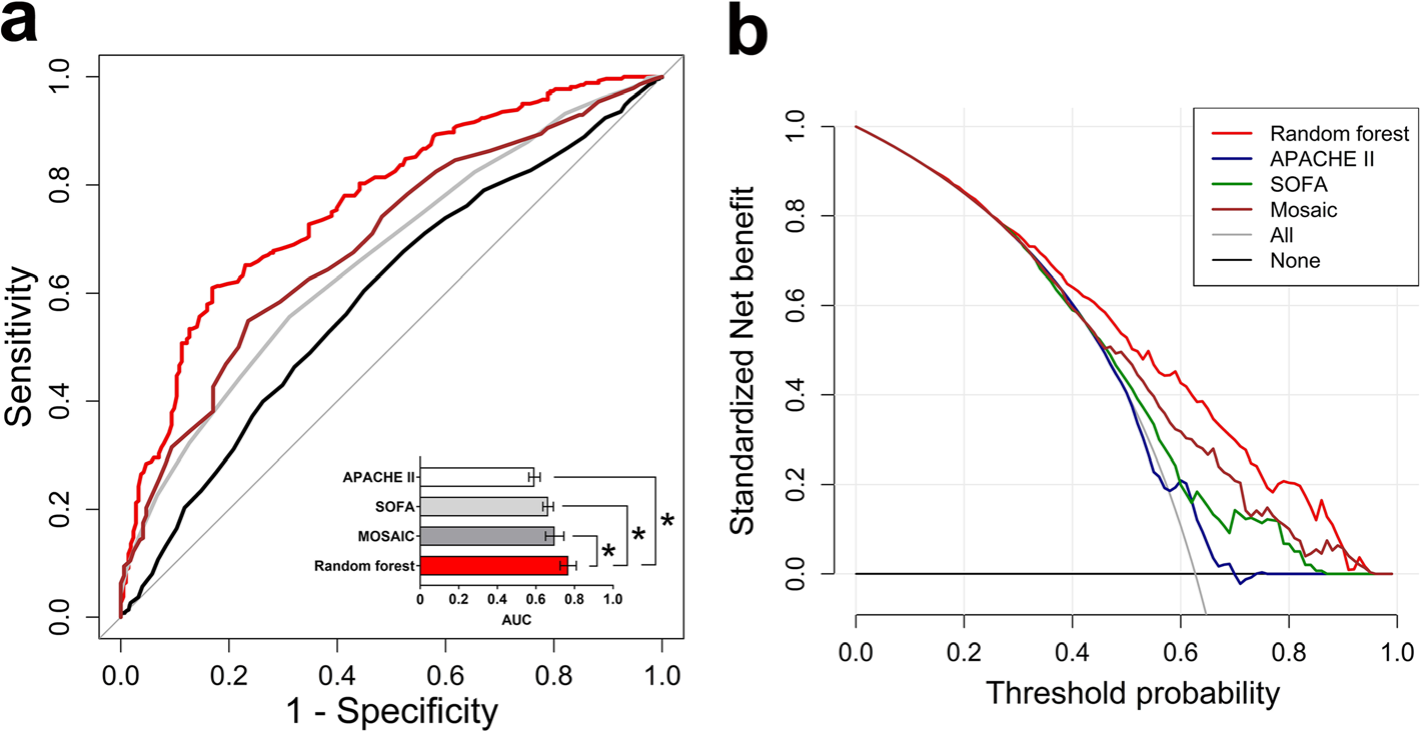
Comparisons of in-hospital mortality prediction models such as random forest, APACHE II, SOFA, and MOSAIC in the test set. **a** Receiver operating characteristic curves of random forest, APACHE II, SOFA, and MOSAIC. The bar graph indicates the median value of the AUC in the model. The error bar indicates the range. **b** Decision curve analysis of random forest, APACHE II, SOFA, and MOSAIC for in-hospital mortality prediction. *P <  0.05. APACHE, Acute Physiology and Chronic Health Evaluation; SOFA, Sequential Organ Failure Assessment; MOSAIC, Mortality Scoring system for AKI with CRRT

The prediction accuracy of in-hospital mortality was further evaluated. A total of 1019 (64.9%) patients died in the hospital. The AUC values of the conventional scoring systems and the machine learning models in the test set are shown in Table 3. The AUCs of the APACHE II, SOFA, and MOSAIC scores were 0.593 (0.563–0.622), 0.664 (0.636–0.691), and 0.690 (0.641–0.740), respectively. The RF model achieved the highest AUC value, 0.768 (0.726–0.810), which was higher than those of APACHE II, SOFA, and MOSAIC scores (Fig. 5a). The net benefit of the RF model ranged from 14 to 95%, which was superior to the conventional scoring systems over the threshold range (Fig. 5b). All of the machine learning models demonstrated better performance than APACHE II and SOFA scores, whereas some of the machine learning models such as RF and ANN had better performance than the MOSAIC model (Table 3). The receiver operating characteristic curves of all of the evaluated models are shown in Additional file 2: Figure S4.

**Table 3:** In-hospital mortality prediction models in the test set

Models	AUC (95% CI)	*P* value*	*P* value^†^	*P* value^‡^	Accuracy	F1 score
APACHE II	0.593 (0.563–0.622)				0.586	0.654
SOFA	0.664 (0.636–0.691)				0.603	0.645
MOSAIC	0.690 (0.641–0.740)				0.633	0.656
κ-Nearest neighbor	0.721 (0.675–0.767)	< 0.001	0.037	0.379	0.673	0.776
Support vector machine	0.755 (0.711–0.799)	< 0.001	< 0.001	0.054	0.686	0.782
Multivariate adaptive regression splines	0.756 (0.713–0.799)	< 0.001	< 0.001	0.050	0.694	0.781
Random forest	0.768 (0.726–0.810)	< 0.001	< 0.001	0.019	0.700	0.757
Extreme gradient boost	0.754 (0.709–0.798)	< 0.001	< 0.001	0.062	0.711	0.790
Artificial neural network	0.762 (0.719–0.806)	< 0.001	< 0.001	0.032	0.707	0.790

*Abbreviations*: *AUC* area under the curve, *CI* confidence interval, *APACHE* Acute Physiology and Chronic Health Evaluation, *SOFA* Sequential Organ Failure Assessment, *MOSAIC* Mortality Scoring system for AKI with CRRT

*Compared with the APACHE II model

^†^Compared with the SOFA model

^‡^Compared with the MOSAIC model

## Discussion

The application of machine learning to medical and clinical conditions forms a major emerging research trend. The present study explores whether applying machine learning could improve the prediction of the mortality of patients who underwent CRRT for AKI. The mortality of these patients has previously been difficult to estimate. The models developed using machine learning algorithms better predicted ICU and in-hospital mortalities than conventional scoring systems such as APACHE II and SOFA, and MOSAIC.

Several scoring systems using clinical and laboratory variables have been developed to predict the outcome of critically ill patients. The APACHE II and SOFA scores are representative of these methods and have demonstrated accurate prediction of mortality in this patient subset [13, 14, 25–28]. However, these approaches showed poor performance for critically ill patients with AKI [11, 12]. Two other scoring models have been applied to critically ill patients with AKI. The HELENICC score, which focused on patients with septic AKI, used five variables (norepinephrine utilization, liver failure, medical condition, and lactate and pre-dialysis creatinine levels) and demonstrated good performance in predicting 7-day mortality (AUC = 0.82) [12]. Another model, which focused on ICU-admitted patients with AKI, also showed good performance for predicting 7-day mortality (AUC = 0.85) [11]. However, these models did not focus on patients initiating CRRT for AKI. A few studies have identified risk factors of mortality in patients receiving CRRT [29, 30]. Nevertheless, it is necessary to develop a mortality prediction model because a few clinical variables may not be sufficient to precisely predict patient outcome. Recently, our MOSAIC model achieved suitable performance with respect to mortality prediction for patients receiving CRRT (AUC = 0.772), but the approach requires further validation and the addition of new variables may be difficult [22]. Machine learning algorithms may solve these problems and will have the added benefit of increased accuracy with the accumulation of data.

Machine learning algorithms have been applied to predict ICU mortality [18, 31–33], although these did not focus on patients undergoing CRRT. In a medical-neurological Indian ICU, the ANN model and APACHE II score achieved similar discriminative power in predicting ICU mortality (AUCs were 0.84 and 0.83, respectively) [31]. Another study developed models for ICU patients with unplanned extubation and found that the RF model achieved the best performance [18]. In the present study, the RF model achieved the highest AUCs for ICU and in-hospital mortalities although there were no significant differences between the RF model and other machine learning models except for the MARS model. The XGB model achieved the highest F1 score. For patients initiating CRRT, the RF and XGB models may be suitable algorithms for predicting mortality.

Decision curve analysis identifies the expected benefit or harm in performing classification at different risk levels. It is useful for comparing models where the default strategies predict all-or-none outcomes such as mortality. This analysis helps to evaluate prognostic models with advantages over other commonly-used models or techniques [23, 24]. This analysis indicated that the RF model improved the net benefit for predicting the ICU mortality and in-hospital mortality compared with APACHE II, SOFA, and MOSAIC scores. Displaying the threshold ranges above the prediction-all and -none curves indicates how the machine learning models will be applicable to clinical practice.

The present study makes several important contributions such as the use of several machine learning models and decision curve analysis according to the specific condition of patients (CRRT). Nevertheless, the present study has some limitations. Because of a single-center design, the models may not be directly applicable to other centers with different treatment plans and patient characteristics. Nevertheless, this issue does not infringe on the purpose of the study, which entails applying machine learning to predict the mortality of patients initiating CRRT for AKI, rather than developing the final generalized model for clinical use. Achieving acceptable performance with a supervised deep-learning algorithm requires more than 5000 data points [34], but the present dataset consisted of a modest sample size. However, the median sample size of the previous 258 studies which used machine learning to analyze ICU data was 488 [20], which is smaller than our sample size. The study identified the most important variables with respect to predicting mortality, but we could not obtain certain degrees of risk, such as the relative risk, which is a common limitation of machine learning algorithms. Concerns could be raised regarding other issues such as overfitting, absence of external validation, and not using fixed time points for the mortality endpoint.

## Conclusion

The mortality of patients who undergo CRRT for AKI has thus far been difficult to estimate. The presented machine learning models predict the mortality of this patient subset better than conventional scoring systems such as APACHE II and SOFA, and MOSAIC. The results indicate that machine learning algorithms are suitable for clinical use in predicting the outcome of patients initiating CRRT for AKI. Future studies will explore whether machine learning is also applicable to predicting other outcomes of the CRRT subset.

## Supplementary information

**Additional file 1: Table S1.** Comparison of baseline characteristics between the training and test sets. **Table S2.** *P* values for differences between machine learning models for ICU mortality prediction in the test set.

**Additional file 2: Figure S1.** Decision curve analysis for predicting ICU mortality in the test set. **a** Random forest. **b** APACHE II. **c** SOFA score. **d** MOSAIC. **e** Total. **Figure S2.** Receiver operating characteristic curves for intensive care unit-mortality-prediction models in the test set. **Figure S3.** Receiver operating characteristic curves for in-hospital mortality-prediction models in the test set.

## Data Availability

Dataset used during the current study is available from the corresponding author on request.

## Abbreviations

AKI: Acute kidney injury
ANN: Artificial neural network
APACHE II: Acute Physiologic Assessment and Chronic Health Evaluation II
AUC: Area under the receiver operating characteristic curve
CI: Confidence interval
CRRT: Continuous renal replacement therapy
ICU: Intensive care unit
KNN: κ-Nearest neighbor
MARS: Multivariate adaptive regression splines
MOSAIC: Mortality scoring system for AKI with CRRT
RF: Random forest
SOFA: Sequential Organ Failure Assessment
SVM: Support vector machine
XGB: Extreme gradient boost
