# Supplementary material for: Machine learning algorithm to predict mortality in patients undergoing continuous renal replacement therapy
Source: Crit Care. 2020 Feb 6;24:42. doi: 10.1186/s13054-020-2752-7 (PMC7006166; doi:10.1186/s13054-020-2752-7)
Supplement: Supplementary file 1 — Additional file 1: Table S1. Comparison of baseline characteristics between the training and test sets. Table S2. P values for differences between machine learning models for ICU mortality prediction in the test set. [file 13054_2020_2752_MOESM1_ESM.docx]

Table S1. Comparison of baseline characteristics between the training and test sets

| Variables | Total  (n = 1,571) | Training  (n = 1,094) | Test  (n = 477) | *P* value |
| --- | --- | --- | --- | --- |
| Age (years) | 63.1 ± 15.2 | 62.6 ± 15.4 | 64.2 ± 14.7 | 0.051 |
| Male (%) | 60.5 | 60.7 | 60.2 | 0.866 |
| Mean arterial pressure (mmHg) | 80.9 ± 17.4 | 81.0 ± 17.3 | 80.7 ± 17.9 | 0.772 |
| Heart rate (/min) | 105.6 ± 25.6 | 105.2 ± 25.4 | 106.6 ± 26.1 | 0.323 |
| Respiratory rate (/min) | 23.9 ± 8.0 | 24.0 ± 8.4 | 23.8 ± 7.0 | 0.628 |
| Body temperature (°C) | 36.3 ± 1.6 | 36.3 ± 1.6 | 36.3 ± 1.6 | 0.303 |
| White blood cells (×10^3^/μL) | 13.8 ± 18.4 | 14.1 ± 20.1 | 13.3 ± 13.7 | 0.414 |
| Hemoglobin (g/dL) | 9.8 ± 2.2 | 9.8 ± 2.2 | 9.7 ±2.1 | 0.638 |
| Blood urea nitrogen (mg/dL) | 50.3 ± 29.3 | 50.3 ± 29.9 | 50.1 ± 27.8 | 0.906 |
| Creatinine (mg/dL) | 2.7 ± 1.7 | 2.7 ± 1.7 | 2.6 ± 1.8 | 0.368 |
| Albumin (g/dL) | 2.7 ± 0.6 | 2.8 ±0.6 | 2.7 ±0.6 | 0.501 |
| pH | 7.3 ± 0.1 | 7.3 ± 0.1 | 7.3 ± 0.1 | 0.322 |
| Sodium (mEq/L) | 138.6 ± 7.9 | 138.6 ± 8.1 | 138.4 ± 7.5 | 0.683 |
| Potassium (mEq/L) | 4.3 ± 0.9 | 4.3 ± 0.9 | 4.3 ± 0.9 | 0.972 |
| Target clearance (ml/min) | 42.5 ± 14.0 | 42.7 ± 14.1 | 42.0 ± 13.8 | 0.375 |
| Diabetes mellitus (%) | 29.0 | 30.3 | 26.2 | 0.116 |
| Hypertension (%) | 28.4 | 27.8 | 29.8 | 0.429 |
| Myocardial infarction (%) | 9.0 | 8.5 | 10.3 | 0.292 |
| Chronic heart failure (%) | 15.4 | 15.2 | 15.9 | 0.704 |
| Stroke (%) | 12.0 | 12.9 | 10.1 | 0.129 |
| Peripheral vascular disease (%) | 7.2 | 7.8 | 5.9 | 0.203 |
| Dementia (%) | 5.2 | 5.2 | 5.0 | > 0.999 |
| Chronic obstructive pulmonary disease (%) | 4.5 | 3.9 | 5.9 | 0.112 |
| Connective tissue disease (%) | 1.5 | 1.4 | 1.9 | 0.503 |
| Peptic ulcer disease (%) | 3.0 | 2.5 | 4.2 | 0.076 |
| Cancer (%) | 36.9 | 36.8 | 36.9 | > 0.999 |
| Ischemic heart disease (%) | 12.4 | 12.1 | 13.2 | 0.560 |
| Chronic kidney disease (%) | 28.8 | 29.7 | 26.6 | 0.226 |
| Ventilator apply (%) | 84.0 | 82.9 | 86.6 | 0.072 |
| Atrial fibrillation (%) | 13.1 | 12.3 | 14.9 | 0.168 |
| PaO_2_/FiO_2_ | 236.6 ± 158.2 | 237.4 ± 160.4 | 234.7 ± 153.1 | 0.756 |
| Anuria (%) | 26.7 | 25.8 | 28.7 | 0.239 |
| ICU mortality | 56.9 | 57.6 | 55.3 | 0.438 |
| In-hospital mortality | 64.9 | 65.8 | 62.7 | 0.250 |

Abbreviations: ICU, intensive care unit.

Table S2. *P* values for differences between the machine learning models for ICU mortality prediction in the test set

| Models | κ-nearest neighbor | Support vector machine | MARS | Random forest | Extreme gradient boost | Artificial neural network |
| --- | --- | --- | --- | --- | --- | --- |
| κ-nearest neighbor | – | 0.479 | 0.648 | 0.062 | 0.640 | 0.632 |
| Support vector machine | 0.479 | – | 0.292 | 0.308 | 0.870 | 0.861 |
| MARS | 0.648 | 0.292 | – | 0.033 | 0.452 | 0.444 |
| Random forest | 0.062 | 0.308 | 0.033 | – | 0.784 | 0.791 |
| Extreme gradient boost | 0.640 | 0.870 | 0.452 | 0.784 | – | 0.992 |
| Artificial neural network | 0.632 | 0.861 | 0.444 | 0.791 | 0.992 | – |

Abbreviations: MARS, multivariate adaptive regression splines.
