# Supplementary material for: Machine learning algorithm to predict mortality in patients undergoing continuous renal replacement therapy
Source: Crit Care. 2020 Feb 6;24:42. doi: 10.1186/s13054-020-2752-7 (PMC7006166; doi:10.1186/s13054-020-2752-7)

Figure S1. Comparisons of intensive care unit mortality-prediction models such as random forest, APACHE II, and SOFA in test data when the discontinuation of CRRT was not censored. **a** Receiver operating characteristic curves of random forest, APACHE II, SOFA, and MOSAIC. The bar graph indicates the median value of the AUC in the model. The error bar indicates the range. **b** Decision curve analysis of random forest, APACHE II, SOFA, and MOSAIC. **P* < 0.05. APACHE, Acute Physiology and Chronic Health Evaluation; SOFA, Sequential Organ Failure Assessment; MOSAIC, Mortality Scoring system for AKI with CRRT.


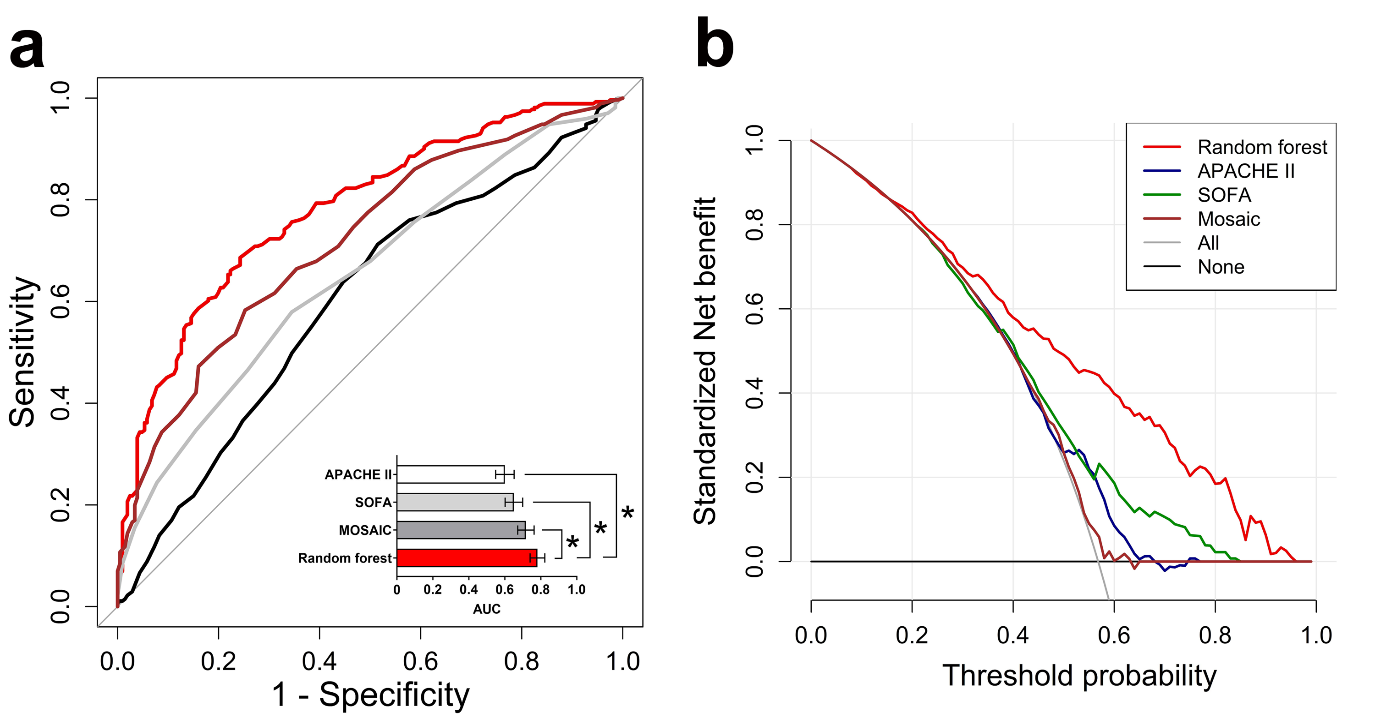


Figure S2. Decision curve analysis for predicting ICU mortality in the test set. **a** Random forest. **b** APACHE II. **c** SOFA score. **d** MOSAIC. **e** Total.

**a** **b**


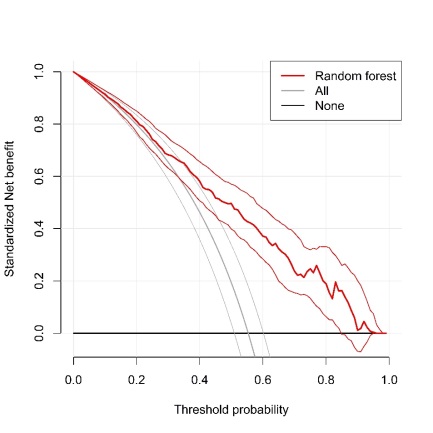

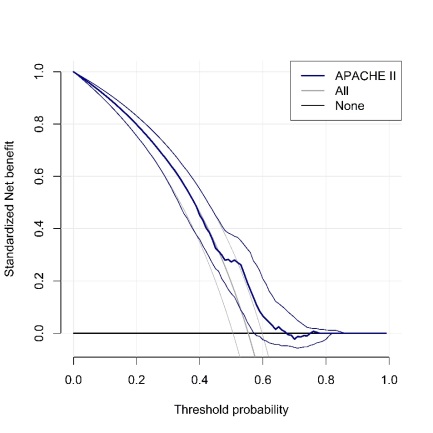


**c d**


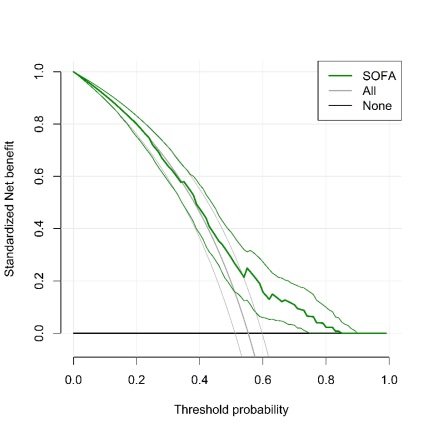


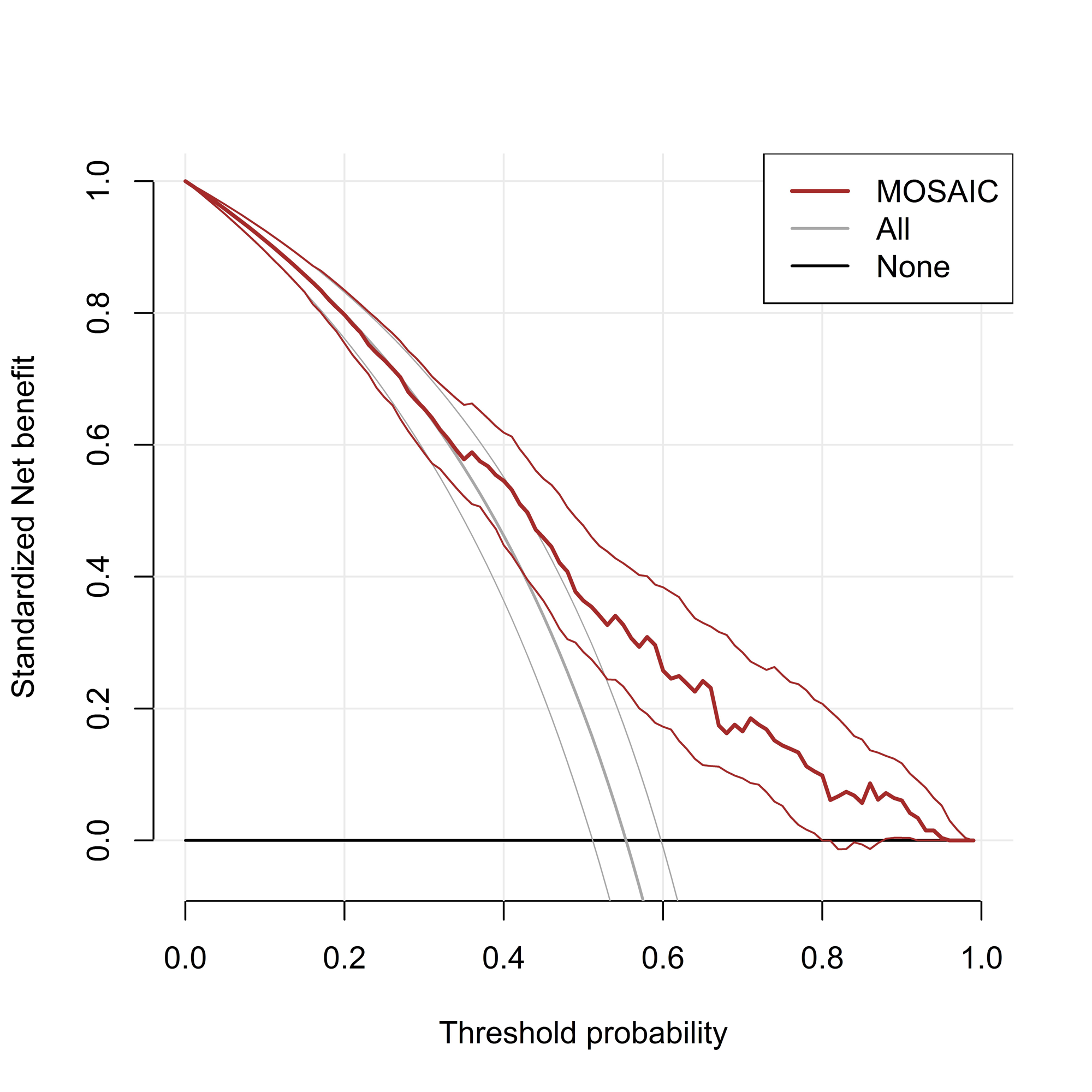


**e**


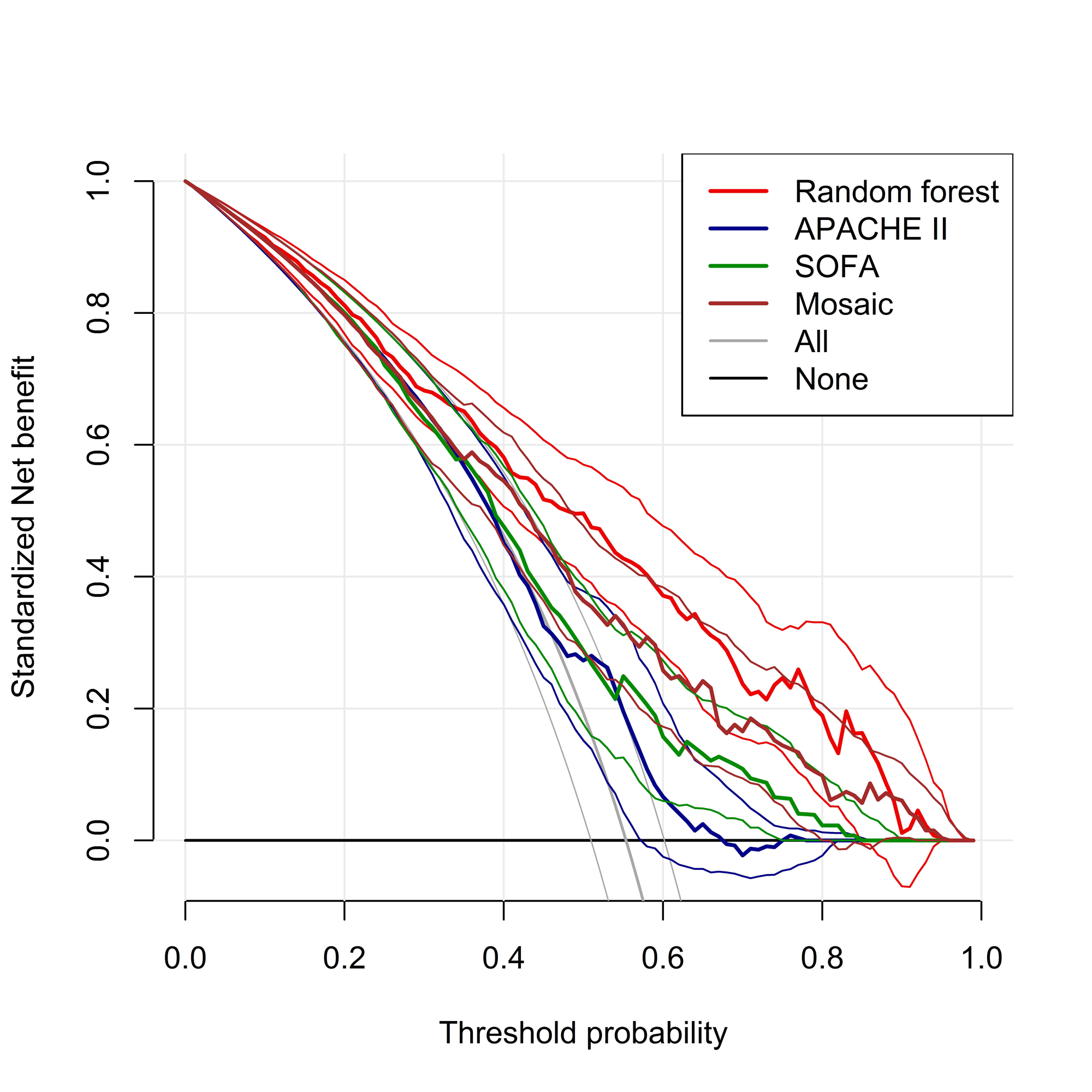


Figure S3. Receiver operating characteristic curves for intensive care unit-mortality-prediction models in the test set.


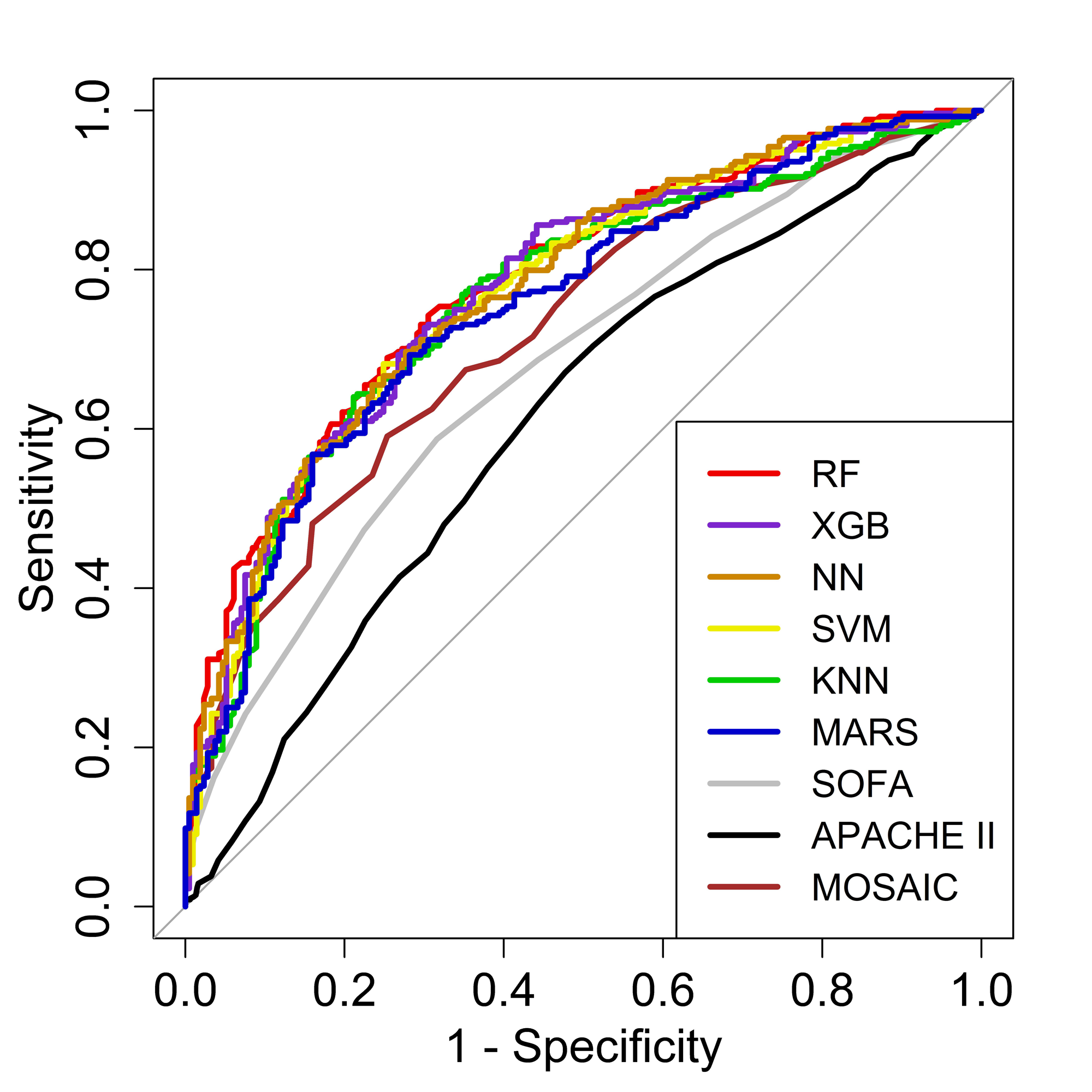


Figure S4. Receiver operating characteristic curves for in-hospital mortality-prediction models in the test set.


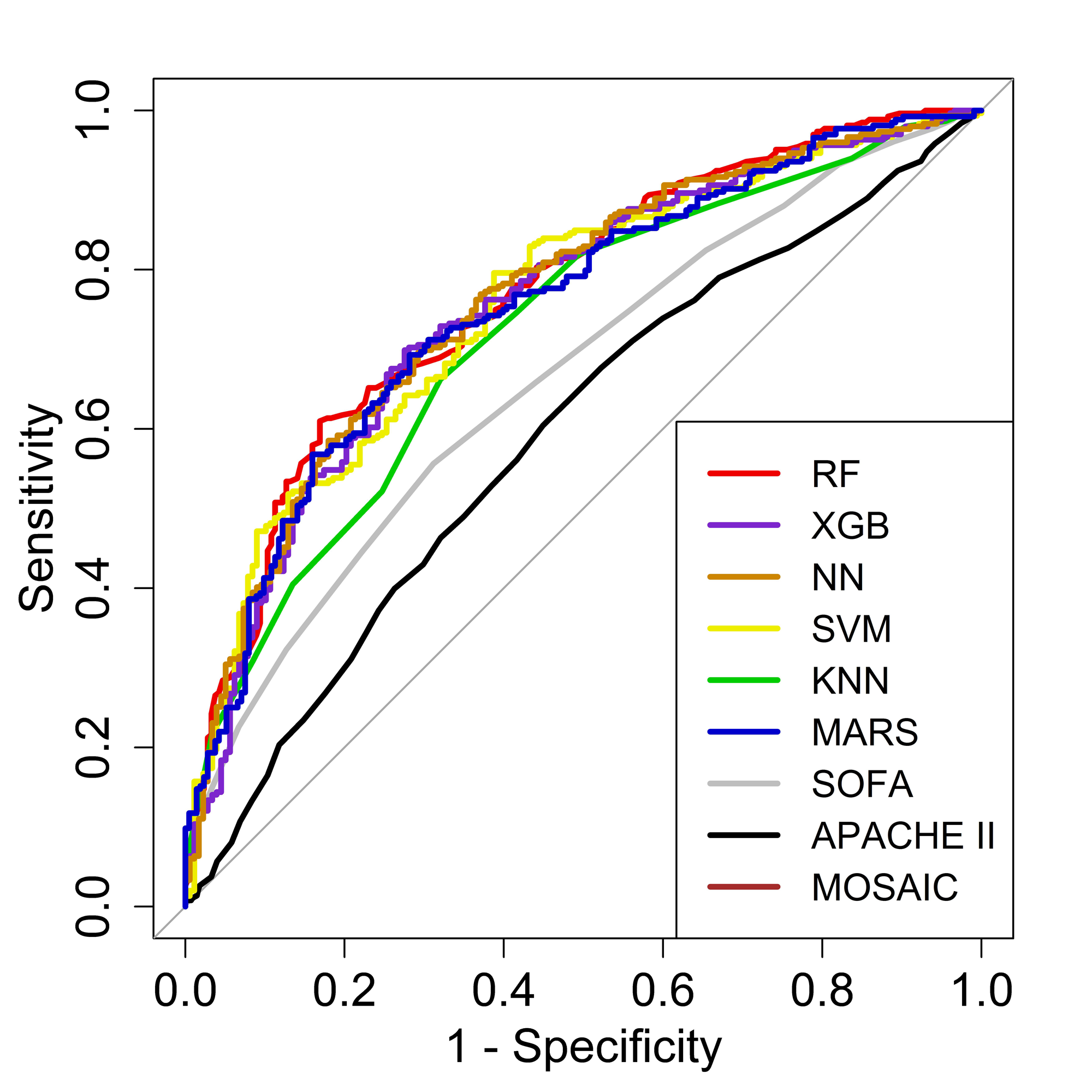

Supplement: Supplementary file 2 — Additional file 2: Figure S1. Decision curve analysis for predicting ICU mortality in the test set. a Random forest. b APACHE II. c SOFA score. d MOSAIC. e Total. Figure S2. Receiver operating characteristic curves for intensive care unit-mortality-prediction models in the test set. Figure S3. Receiver operating characteristic curves for in-hospital mortality-prediction models in the test set. [file 13054_2020_2752_MOESM2_ESM.docx]
